# Use of sodium oxybate for the treatment of alcohol withdrawal syndrome in patients with acute alcohol-associated hepatitis: A 4-patient case report

**DOI:** 10.1097/MD.0000000000039162

**Published:** 2024-08-02

**Authors:** Fabio Caputo, Alberto Casabianca, Camilla Brazzale, Lisa Lungaro, Anna Costanzini, Giacomo Caio, Roberto De Giorgio, Gianni Testino, Fabio Piscaglia, Paolo Caraceni

**Affiliations:** a Centre for the Study and Treatment of Alcohol-Related Diseases, Department of Translational Medicine, University of Ferrara, Ferrara, Italy; b Department of Internal Medicine, SS Annunziata Hospital, University of Ferrara, Cento (Ferrara), Italy; c Department of Translational Medicine, University of Ferrara, Ferrara, Italy; d Unit of Addiction and Hepatology, ASL3 c/o Ospedale Policlinico San Martino, Genova, Italy; e Division of Internal Medicine, Hepatobiliary and Immunoallergic Diseases, IRCCS Azienda Ospedaliero-Universitaria di Bologna, Bologna, Italy; f Department of Medical and Surgical Sciences, University of Bologna, Bologna, Italy; g Department of Internal Medicine and Surgery (DIMEC), Alma Mater Studiorum, University of Bologna, Bologna, Italy; h Unit of Semeiotics, Liver and Alcohol-Related Diseases, IRCCS Azienda Ospedaliero-Universitaria of Bologna, Bologna, Italy.

**Keywords:** acute alcohol-associated hepatitis, alcohol withdrawal syndrome, case report, sodium oxybate

## Abstract

**Introduction:**

During the treatment of alcohol use disorder, alcohol withdrawal syndrome (AWS) can occur. Benzodiazepines remain the “gold standard” for the pharmacological treatment of AWS. However, other drugs have been approved in some European Countries for the treatment of AWS: namely, clomethiazole in Spain and Germany and sodium oxybate in Italy and Austria. Acute alcohol-associated hepatitis (AAH) is a distinct clinical syndrome characterized by the recent onset of jaundice with or without other signs of liver decompensation in patients with ongoing alcohol consumption.

**Rationale:**

We report 4 paradigmatic clinical cases to analyze the efficacy, safety, and tolerability of the very short half-life (30–45 minutes) sodium oxybate (SO) in the management of AWS with moderate to severe AAH. Compared to SO, “as needed” short-acting benzodiazepines, currently prescribed to treat AWS in patients with AAH, have a much longer half-life (5–25 hours) which increases the risk of drug accumulation. The very short half-life of SO provides a fixed dose approach allowing for a more effective control of AWS than “as needed” therapy throughout the 24 hours.

**Patient concerns:**

Patients reported anxiety, agitation, diffuse abdominal pain, loss of appetite, and nausea with elevation in serum bilirubin and 2 of them had abdomen distension due to ascites.

**Diagnosis:**

Patients were affected by moderate or severe AWS and moderate or severe AAH on alcohol-related liver cirrhosis.

**Interventions:**

In order to suppress AWS, all patients were treated with oral sodium oxybate at a dose of 25 mg/kg/day, progressively increased to 50 to 100 mg/kg/day, divided into 3 to 5 administrations.

**Outcomes:**

SO was efficient, safe and tolerable in suppressing AWS even in patients with severe AAH. All treated patients showed a rapid improvement of all symptom (via the Clinical Institute of Withdrawal Assessment for Alcohol Scale) and liver test scores (Model for End-Stage Liver Disease).

**Conclusion:**

Because of its short half-life, SO can be considered a safe and effective pharmacological option for the AWS in patients with moderate to severe AAH even in comparison to short-acting benzodiazepines, thus avoiding the risk of accumulation. Notably, SO guarantees a fixed approach to cover the possible onset of AWS throughout the 24 hours.

## 1. Introduction

Alcohol use disorder (AUD) shows a worldwide prevalence of 20% to 30% and 10% to 15% in men and women, respectively, and may be mild, moderate or severe.^[1]^ During the treatment of AUD, conditions such as acute alcohol intoxication or alcohol withdrawal syndrome (AWS) can occur, requiring a specific approach.^[2]^ In fact, about 50% of AUD sufferers may have symptoms of AWS (i.e., increased hand tremor, nausea or vomiting, psychomotor agitation) when they reduce or discontinue their alcohol consumption.^[2]^ AWS is monitored using an easy-to-administer, repeatable and sensitive measuring scale: the Clinical Institute of Withdrawal Assessment for Alcohol Scale (CIWA-Ar). The CIWA-Ar scale examines the intensity of 10 parameters: nausea and vomiting, agitation, anxiety, hearing disorders, visual disorders, sensory alterations, headache, sweating, tactile disorders and tremors.^[2]^

Benzodiazepines (BDZs) remain the “gold standard” for the pharmacological treatment of AWS.^[2]^ Oxazepam and lorazepam are considered the drug of choice for the treatment of AWS in patients with acute or chronic alcohol-related liver disease.^[2]^ However, other drugs have been approved in some European Countries for the treatment of AWS: namely, clomethiazole in Spain and Germany and sodium oxybate in Italy and Austria.^[3–6]^ Sodium oxybate (SO), also called gamma-hydroxybutyric acid, is a short-chain fatty acid, physiologically detectable in the central nervous system, particularly in the thalamus, the hypothalamus, and the basal ganglia.^[4]^ SO is structurally similar to the GABA neurotransmitter and binds to the GABA_B_ receptor. A Cochrane meta-analysis showed that, at a dose of 50 mg/kg divided into 3 to 6 daily administrations, SO is as effective as BDZs (diazepam and oxazepam) and clomethiazole in reducing the alcohol withdrawal related symptoms.^[3,4]^ One case report, evaluating SO in a patient with end-stage alcohol-related liver disease with ascites, showed that the drug is safe and effective in the treatment of AWS.^[2,4]^

Acute Alcohol-associated Hepatitis (AAH) is a distinct clinical syndrome characterized by the recent onset of jaundice with or without other signs of liver decompensation [i.e., ascites or hepatic encephalopathy (HE)] in patients with ongoing alcohol consumption of more than 3 drinks (approximately 40 g) per day for women and 4 drinks (approximately 50–60 g) per day for men for 6 months or more.^[5,7]^ The laboratory profile of AAH reveals neutrophilia, hyperbilirubinemia (more than 3 mg per deciliter), and serum levels of AST over twice the upper limit in the normal range, with an AST/ALT ratio typically above 1.5 to 2.0 with both values <400 IU per liter. Moderate AAH occurs frequently, and its incidence is probably underestimated compared to its severe form.^[5,7]^ In severe manifestations of AAH, prolonged prothrombin time, hypoalbuminemia, and decreased platelet count are frequently observed.^[5,7]^ Current models used to predict short-term mortality in severe AAH include the Model for End-Stage Liver Disease (MELD) score; today’s guidelines recommend its use for patient risk stratification, and corticosteroids remain the drug of choice.^[5,7]^ The Lille score is a dynamic means of assessing prognosis and identifying nonresponse to corticosteroids; usually calculated at day 7, alternatively it may be calculated at day 4.^[5,7]^

Based on the daily practice and referrals to our Center, we report 4 paradigmatic clinical cases to analyze the efficacy, safety and tolerability of SO in the management of AWS with moderate and severe AAH. All patients were admitted to the Internal Medicine Unit of the “SS. Annunziata” Hospital in Cento (Ferrara, Italy) where the Center for the Study and Treatment of Alcohol-Related Diseases is located.

## 2. Case presentation

### 2.1. Case 1

A 53-year-old man was admitted in November 2018. The patient had a medical history of alcohol-associated liver cirrhosis (ALC), ischemic heart disease with previous coronary stent placement, arterial hypertension, smoking habit, and a history of severe AUD for approximately 3 years with an intake of large amounts of alcohol (12–14 units per day) over 6 to 8 months. For a few days the patient had been complaining of asthenia, diffuse abdominal pain, diarrhea, loss of appetite with nausea and vomiting in the morning. The patient appeared alert and cooperative. Vital parameters were within normal limits. Laboratory tests carried out on admission are shown in Table 1. Viral and autoimmune hepatitis were excluded. An abdominal ultrasound showed an uncomplicated ALC.

**Table 1:** Scores and laboratory findings on admission and discharge of the 4 patients.

	Case 1		Case 2		Case 3		Case 4	
	Admission	Discharge	Admission	Discharge	Admission	Discharge	Admission	Discharge
CIWA-Ar	14	3	12	3	20	3	20	3
MELD	15	10	15	15	27	24	21	16
mDF	11.7	6.4	17.2	19.9	76.7	53.9	36.7	21.6
Child-Pugh	8	6	9	8	12	12	12	10
MCV (fl)	88	88	101	104	101	100	94	108
GGT (U/L)	2901	953	200	159	131	120	693	405
AST (U/L)	547	125	64	84	453	95	189	54
ALT (U/L)	173	86	42	46	136	67	20	30
Total Bil. (mg/dL)	7.12	1.84	2.63	3.49	10.36	10.37	7.23	4.47
Direct Bil. (mg/dL)	3.81	0.90	1.22	1.34	5.76	4.41	3.60	1.89
Albumin (g/L)	30.1	31.1	30.5	34.8	25.7	25.7	22.5	27.2
Creatinine (mg/dL)	0.58	0.58	0.42	0.42	0.64	0.54	0.38	0.40
Ammonium (µmol/L)	109	53	84	84	70	106	59	54
INR	1.12	1.20	1.35	1.44	2.97	2.24	1.83	1.40

**Figure 1.**
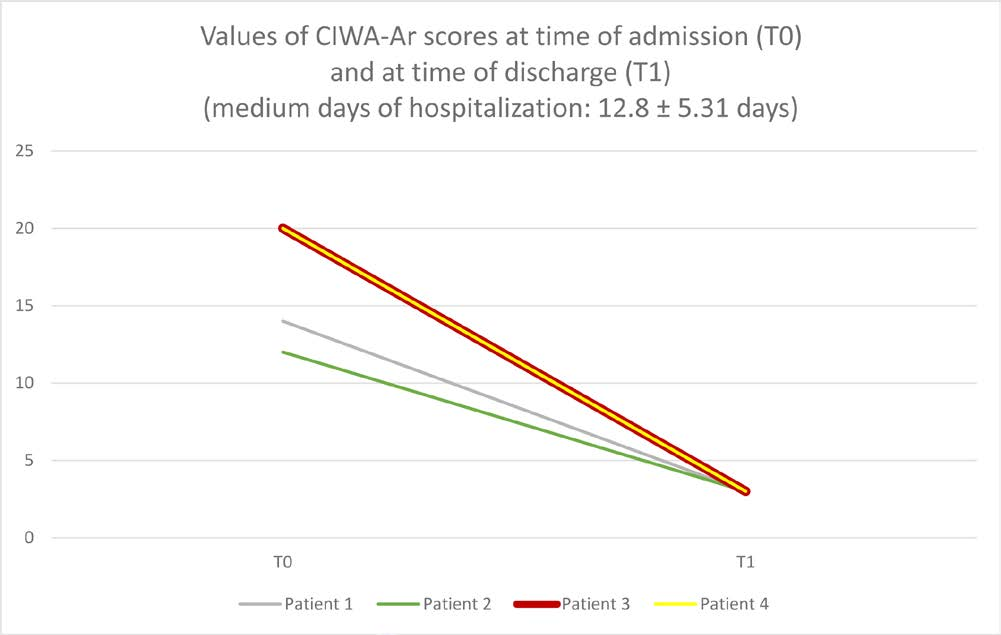
Modifications of CIWA-Ar scores during the period of hospitalization in the 4 patients.

The patient presented moderate AWS with CIWA-Ar of 14 (Fig. 1). Oral SO at a dose of 25 mg/kg/day, progressively increased within 1 day to 50 mg/kg/day, divided into 4 administrations was commenced. Withdrawal symptoms were well controlled and the CIWA-Ar at discharge was 3 (Fig. 1).

In association with AWS, the laboratory picture and clinical conditions indicated a moderate AAH, with MELD score of 15. Beside SO, the patient did not require steroid treatment and was treated exclusively with supportive therapy, specifically i.v. hydration and thiamin (200 mg i.m. per day for the first 3 days followed by 300 mg orally) in order to prevent the onset of Wernicke-Korsakoff syndrome.^[2,8]^ During hospitalization, the patient displayed regression of all symptoms. Laboratory values at discharge are shown in Table 1. In addition to the chronic pharmacological treatment of his heart disease, the patient was discharged with oral acamprosate 666 mg t.i.d. (a common therapeutic regimen in patients with >60 kg of body weight) to maintain alcohol abstinence.^[8,9]^

### 2.2. Case 2

A 52-year-old man was admitted in January 2019. The patient had a history of i.v. substance addiction (heroin), both alcohol and HCV-related liver cirrhosis (the HCV infection was successfully treated in 2012), type 2 diabetes, arterial hypertension, hypothyroidism, lithiasis of the gallbladder, and severe AUD; in the previous 8 to 10 months he had relapsed into alcohol misuse (8–10 units per day). However, upon admission the patient was in acceptable clinical condition, with vital parameters in the normal range. Laboratory tests at admission are shown in Table 1. Viral and autoimmune hepatitis were excluded; only anti-HCV antibodies were positive due to the prior HCV infection. The abdominal ultrasound scan showed a picture of micro- and macronodular ALC, in the absence of ascitic effusion.

The AWS was moderate with a CIWA-Ar on admission of 12 points (Fig. 1). The patient was treated with oral SO starting from 25 mg/kg/day progressively increased within 1 day to 50 mg/kg/day divided into 3 administrations. The withdrawal symptoms were well controlled and suppressed, with a CIWA-Ar at discharge of 3 (Fig. 1).

In light of the medical history and the laboratory test values on admission, the patient was diagnosed with moderate AAH and MELD score of 15. To prevent the onset of Wernicke-Korsakoff syndrome, oral thiamin (200 mg i.m. per day for the first 3 days followed by 300 mg orally) was commenced.^[2,8]^

The clinical picture improved significantly while the laboratory parameters improved only partially (Table 1). The patient was discharged with oral baclofen 10 mg t.i.d. to maintain alcohol abstinence.^[8,9]^

### 2.3. Case 3

A 65-year-old woman was admitted in September 2020. The patient had a history of ALC, previous smoking habit, hysterectomy, hemorrhoidal disease, and moderate AUD. She had consumed large amounts of alcohol (8–10 units per day) for at least 8 to 10 months. She recently reported an increase in abdominal circumference accompanied by significant elevation of the serum bilirubin levels. On admission, the patient initially appeared alert, spatially aware and cooperative; the abdomen was prominent due to ascites. She did not show signs indicative of HE. Laboratory tests at admission are shown in Table 1. Viral and autoimmune hepatitis were excluded.

The patient presented severe withdrawal symptoms, with CIWA-Ar score of 20 (Fig. 1). At this point, we set up a therapy with oral SO starting from 25 mg/kg/day progressively increased within 2 days to 50 mg/kg/day and to 100 mg/kg divided into 3 daily administrations. In addition, i.v. lorazepam 4 mg was prescribed as needed, but administered only once during the hospitalization. AWS was well controlled, with a CIWA-Ar at discharge of 3 (Fig. 1).

The laboratory values were markedly altered, outlining a picture of severe AAH, resulting in acute-on-chronic liver failure (ACLF). The MELD score was 27, and corticosteroid therapy with oral prednisone 40 mg/day was started.^[7,8]^ Oral thiamin (200 mg i.m. per day for the first 3 days followed by 300 mg orally) to prevent the onset of Wernicke-Korsakoff syndrome was commenced.^[2,8]^ Because of moderate ascites (grade 2), a diagnostic paracentesis was performed. The white count performed on the ascitic fluid showed polymorphonucleates count of 50/mm^3^ ruling out spontaneous bacterial peritonitis. The dose of diuretic therapy with oral potassium canrenoate was increased up to 400 mg/day, and oral furosemide therapy was also concurrently added up to 100 mg/day. Two days later, following the onset of HE, diuretic therapy was discontinued and oral lactulose 20 g t.i.d. and rectal lactulose 200 g b.i.d. were administered in association with oral rifaximin 400 mg t.i.d. Patient’s response to steroid therapy was assessed using the Lille score after 7 days of treatment: the score resulted to be 0.73, indicating nonresponse, and accordingly steroid treatment was discontinued. There was no significant improvement in laboratory values, and the MELD score was 24 (Table 1). The ACLF picture persisted without any improvement after 7 days of therapy. The patient was eventually referred to the Transplant Unit of St. Orsola-Malpighi Hospital in Bologna to evaluate the feasibility for Orthotopic Liver Transplantation.^[7,8]^ At the time of discharge from our ward, in light of the onset of an overt HE and the well-controlled AWS, oral SO was discontinued while oral lactulose 20 g t.i.d. and rectal lactulose 200 g b.i.d. were continued in association with oral rifaximin 400 mg t.i.d. In October 2020 the patient was transplanted and she is still in good clinical condition.

### 2.4. Case 4

A 46-year-old woman was admitted in December 2019. The patient had a history of severe AUD with alcohol intake of 8 to 10 units per day for at least 1 year, ALC, erosive gastritis with reflux esophagitis and F1 esophageal varices, hiatal hernia, septal, and centrilobular emphysema related to cigarettes smoking. The admission to the hospital was due to abdominal pain with moderate grade ascites on abdominal ultrasound. On admission, the patient appeared alert, spatially aware and cooperative; the abdomen was distended due to the presence of moderate ascites. Laboratory tests are shown in Table 1. Viral and autoimmune hepatitis were excluded.

The patient presented severe withdrawal symptoms with CIWA-Ar of 20 (Fig. 1). We initiated AWS therapy with oral SO starting from 25 mg/kg/day and progressively increased within 2 days to 50 mg/kg/day and to 100 mg/kg divided into 5 daily administrations. Since AWS was not fully controlled by the administration of SO, after 2 days of hospitalization, it was necessary to add BDZ therapy with i.v. lorazepam administered initially at a dose of 2 mg 4 times a day, then orally 2.5 mg 3 times a day, and progressively tapered down to 2.5 mg per day till discontinuation upon discharge. AWS was well controlled, with a CIWA-Ar of 3 at discharge (Fig. 1).

Laboratory tests and clinical conditions presented a picture of severe AAH, resulting in ACLF, with MELD score of 21. Due to the patient’s compromised condition, steroid therapy was started with oral prednisone 40 mg/day plus metadoxine 500 mg t.i.d.^[7–9]^ On day 7 the steroid treatment showed efficacy as indicated by the Lille score (0.279). The treatment with oral prednisone 40 mg/day plus metadoxine t.i.d. was continued for 21 further days, completing a total of 28 days of therapy; afterwards, the steroid treatment was tapered and discontinued, and metadoxine was stopped. Likewise, the other reported cases, also this patient was supplemented with oral thiamin (200 mg i.m. per day for the first 3 days followed by 300 mg orally) to prevent the onset of Wernicke-Korsakoff syndrome.^[2,8]^ The ascites found on admission was treated with oral potassium canrenoate at a dose of 200 mg/day, then reduced to 100 mg/day at discharge. The laboratory picture improved significantly at discharge, and the MELD score was 16 (Table 1).

The patient was discharged in fair clinical conditions, with the following therapy: oral prednisone 40 mg/day, oral potassium canrenoate 100 mg/day, and oral metadoxine 500 mg t.i.d. In addition, oral acamprosate 666 mg in the morning and 333 mg in the afternoon and evening (a common therapeutic dosage in patients with <60 kg of body weight) was prescribed.^[8,9]^

## 3. Discussion

These are the first case report of 4 patients showing efficacy, safety and tolerability of oral SO in the treatment of moderate to severe AWS in patients with moderate AAH and severe AAH with ACLF.

Indeed, patients rapidly improved from all symptoms of AWS as demonstrated by the progressive reduction of CIWA-Ar score. CIWA-Ar test is useful in monitoring the trend of AWS as 3 different scores of the test can effectively identify different intensities of alcohol withdrawal symptoms (mild: <8 points; moderate: 8–15 points; severe >15 points).^[2,8]^ We used an oral dose of SO ranging from 25 to 100 mg/kg/day, not exceeding the usual prescription dose. Following the indication of oral SO administration for ALC, patients were treated initially with 25 mg/kg/day then titrated up to 100 mg/kg/day, when necessary.^[10]^ However, in patient 3 and 4, both on the highest dose of oral SO (100 mg/kg/day), the occasional use of i.v. lorazepam or i.v. lorazepam at a fixed dose regimen was commenced.^[2]^ At discharge, oral SO was discontinued without any tapering dose, while lorazepam in patient 4 was tapered down until discontinued because of the addictive property of BDZs.^[2]^ Taking into account the association of oral SO and lorazepam, patient 4 did not show any sedative effects, hence avoiding the single use of lorazepam at high doses (4–10 mg t.i.d.) a commonly used therapeutic strategy in severe forms of AWS,^[2,6]^ although hampered by major sedative effects. In patients 3, and 4 metadoxine was used. The use of metadoxine in association with steroid therapy in patients with severe AAH is able to reduce the mortality rate after 1 month compared to steroid therapy alone.^[9]^ In order to maintain abstinence from alcohol, acamprosate, and baclofen were commenced; these are the drugs of choice to maintain abstinence from alcohol in patients with liver impairment.^[8,9]^

Moreover, all patients (whereas patient 3 to a lower extent) had improved liver function at discharge. Although oral SO is metabolized by the liver, it appeared to be safe for patients with liver impairment due to its very short half-life (30–45 minutes).^[2,4,8,10]^ The pharmacokinetic profile of SO has been shown to be similar in patients with ALC with or without ascites^[10]^ confirming the safety of this drug in patients with liver impairment. Short acting BDZs (lorazepam and oxazepam) are considered the gold standard for the treatment of AWS in patients with AAH and in patients with ALC with an ‘as-needed’ pharmacological approach, yet their half-life (5–25 hours) is longer than SO.^[2]^ Hence, the very short half-life of SO provides a fixed dose approach to SO allowing more effective control of alcohol withdrawal symptoms than “as needed” therapy throughout the 24 hours.

Furthermore, none of the reported patients experienced side effects due to oral SO. This is in line with the literature indicating that only 10% of patients suffer from side effects during SO treatment. In addition, no case of craving and/or drug abuse was recorded in the reported patients. Craving and abuse remain a controversial issue during the administration of SO although it is very rare during clinical trials and in everyday clinical administration.^[4]^ All patients in our case series were hospitalized and the risk of abuse was minimized by physicians and nurses monitoring drug administration, and SO was discontinued just before discharge.

Although AWS in cases with AAH is often associated with severe complications (e.g., infections), all patients in this case series are alive and none of them required major supportive measures, such as mechanical ventilation and/or intensive care unit admission. This positive outcome may be ascribed to the oral administration of SO which was able to contribute to a prompt control of AWS. Recent evidence demonstrated that the intravenous use of BDZs for AWS treatment was independently associated with higher mortality compared to oral BDZs.^[3]^ In addition, patient 4 had overt HE. It is well-known that some symptoms of HE may overlap with those of AWS and acute alcohol intoxication.^[2,8]^ However, in patient 4 overt HE was developed as a consequence of the ACLF-related liver function worsening, at a time when AWS was already suppressed by SO. Moreover, when HE occurred, SO was immediately discontinued. Indeed, GABAergic agents are not indicated in patients with overt HE due to the possible worsening of this clinical condition.^[8]^

These case reports show some limitations. First, the absence of a control group using short-acting BDZs. Although SO has already shown to exhibit the same efficacy of the short-acting BDZ oxazepam in the treatment of AWS,^[2,4]^ studies comparing SO and BDZs in patients with AWS and liver impairment are eagerly awaited. Second, as a safety feature, BDZs remain the only medications able to prevent more severe complications of AWS (e.g., delirium tremens and seizures), but they may increase the risk of sedation particularly in patients with liver failure.^[2]^ In contrast, due to its very short half-life, SO may present a reduced risk of sedation in patients with liver impairment.

## 4. Conclusion

In conclusion, due to its very short half-life even in comparison to short-acting benzodiazepines, SO can be considered a safe, efficient and tolerable pharmacological option for the treatment of moderate to severe AWS in patients with moderate and severe AAH including cases complicated by ACLF. SO can be useful to reduce the risk of BDZ accumulation. In addition, with its fixed approach, SO prevents possible onset of AWS throughout the 24 hours. Based on the preliminary data from this small case series, a controlled clinical trial testing the efficacy, safety and tolerability of oral SO compared to short-acting BDZs for the treatment of AWS in patients with AAH and ALC is warranted.

## Author contributions

**Conceptualization:** Fabio Caputo.

**Investigation:** Alberto Casabianca, Camilla Brazzale, Gianni Testino.

**Supervision:** Roberto De Giorgio.

**Visualization:** Anna Costanzini, Giacomo Caio, Fabio Piscaglia, Paolo Caraceni.

**Writing – original draft:** Fabio Caputo, Camilla Brazzale.

**Writing – review & editing:** Fabio Caputo, Lisa Lungaro, Roberto De Giorgio, Gianni Testino.
